# Mosquito Surveillance in Quarantine Areas of Ports and Airports in the Chungcheong Region, Republic of Korea, 2025

**DOI:** 10.56786/PHWR.2026.19.42.3

**Published:** 2026-11-05

**Authors:** Beoulhui Lim, Youngsil Yoon, Jun Hyeong Jang, Soyeon Kim, Bo-Ram Kim, Jun Ho Jeon

**Affiliations:** Division of Laboratory Diagnosis and Analysis, Chungcheong Regional Center for Disease Control and Prevention, Korea Disease Control and Prevention Agency Daejeon Korea

**Keywords:** Mosquito-borne diseases, Vector surveillance, Flavivirus, Quarantine

## Abstract

**Objectives:**

To investigate mosquito species composition and mosquito-borne pathogens in airport and seaport quarantine areas in the Chungcheong region to support the prevention of imported vectors and mosquito-borne diseases.

**Methods:**

Mosquitoes were collected twice monthly from March to November 2025 at nine sites in Gunsan Port, Pyeongtaek-Dangjin Port, Daesan Port, and Cheongju International Airport, using black light, Biogents-sentinel, and gravid traps, except once in March and November. Collected mosquitoes were identified and subsequently tested for flaviviruses and chikungunya virus using nucleic acid detection assays.

**Results:**

A total of 8,393 mosquitoes representing nine species were collected. *Culex pipiens* predominated (67.3%), followed by *Aedes albopictus* (16.9%), *Aedes vexans* (4.0%), *Anopheles* spp. (3.4%), and *Ochlerotatus koreicus* (2.8%). Mosquito abundance peaked in July, with Gunsan Port accounting for 58.8% of all collections. All mosquito pools tested negative for flavivirus and chikungunya virus.

**Conclusions:**

No exotic mosquito species, including *Aedes aegypti*, or mosquito-borne pathogens were detected in the quarantine zones. However, *Cx. pipiens*, a potential vector of West Nile virus, and *Ae. albopictus*, a potential vector of dengue, Zika, and chikungunya viruses, increased by 152.7% and 52.3%, respectively, compared with the previous year (2,235 and 929, respectively). Continued mosquito vector surveillance in quarantine areas can provide baseline data to support for early detection of introduced mosquito-borne disease risks and inform effective quarantine and vector control strategies.

**Key messages**

**① What is known previously?**

Mosquitoes transmit various infectious diseases, and the risk of imported mosquito-borne diseases is increasing with international travel and climate change. Indigenous mosquitoes in the Republic of Korea can also transmit these pathogens.

**② What new information is presented?**

In 2025, 8,393 mosquitoes representing nine species were collected from quarantine areas in the Chungcheong region. *Culex pipiens* predominated, and all samples tested negative for flaviviruses and chikungunya virus.

**③ What are implications?**

Continuous vector surveillance in quarantine areas provides essential data for early detection of imported mosquito-borne diseases, risk assessment, and effective quarantine and vector control.

## Introduction

Recently, with the increase in international exchange and overseas travel, the risk of imported infectious diseases in the Republic of Korea (ROK) continues to increase. In particular, mosquito-borne infectious diseases, such as dengue fever, chikungunya fever, Zika virus infection, and West Nile fever, can be transmitted between countries through the movement of infected individuals and vectors and are recognized as major threats to international public health [1,2].

Climate change is known to be a major factor affecting the occurrence and spread of mosquito-borne infectious diseases. Rising temperatures and changes in precipitation patterns affect mosquito survival, reproduction, and seasonal patterns of occurrence, and can lead to expansion of the habitat range and changes in the occurrence timing of some mosquito species [3,4]. Accordingly, the World Health Organization (WHO) emphasizes the importance of strengthening surveillance systems and continuous vector management for the prevention of mosquito-borne infectious diseases [2].

In the ROK, imported dengue fever cases continue to be reported [5]. Thus, the risk of imported mosquito-borne infectious diseases in the ROK persists [6]. Chikungunya fever and dengue fever are known to be transmissible by *Aedes albopictus*, which also inhabits the ROK, further increasing the importance of continuous evaluation of the possibility of importation of diseases through infected overseas travelers and imported vectors, as well as imported vector surveillance in quarantine areas [7].

WHO recommends strengthening vector surveillance capacity in areas where international exchange occurs, such as airports and ports [2]. In the ROK, vector surveillance projects for infectious diseases are also being conducted in quarantine areas in accordance with the Quarantine Act and quarantine work guidelines. Efforts are being continued to detect the inflow of imported infectious diseases into the ROK at an early stage and prevent their spread.

The Chungcheong region is an area where major international exchange hubs, such as Cheongju International Airport, Gunsan Port, Pyeongtaek-Dangjin Port, and Daesan Port, are located, and has a high possibility of inflow of mosquito-borne imported infectious diseases and infectious disease vectors. Therefore, this study aimed to analyze the species distribution and seasonal patterns of occurrence of mosquitoes collected in the quarantine areas of the Chungcheong region in 2025, and investigate the carriage of flaviviruses and chikungunya virus (CHIKV) in mosquitoes to evaluate the risk of inflow of imported infectious disease vectors in the quarantine areas and to establish a scientific basis for establishing future vector surveillance strategies in the quarantine areas.

## Methods

### 1. Mosquito Collection

For the surveillance of imported infectious disease vector mosquitoes and pathogens in mosquitoes, mosquito collection was conducted twice a month from March to November (once a month in March and November each) at nine sites in the quarantine areas of the Chungcheong region (four sites at Gunsan Port, two sites at Pyeongtaek-Dangjin Port, two sites at Daesan Port, and one site at Cheongju International Airport) (Tables 1, 2). The collection sites were selected in environments with high human traffic, such as cargo yards or traveler movement paths within the quarantine areas, having sewers or standing water, and with trees and shrubs. Black light traps (UV-LED Black hole plus; Bio-Trap) Biogents (BG)-sentinel traps (BG-sentinel trap; Biogents), and gravid traps (CDC gravid trap; John W.Hock Company) were used as equipment for mosquito collection (Table 1). In the vacant lot near the Daesan branch office building, the gravid trap was installed at a distance of 10 m or more to prevent the BG-sentinel trap and gravid trap from affecting each other. At Cheongju International Airport, black light traps were used until April, and BG-sentinel traps were used starting in May. Dry ice and lures were used as mosquito attractants. To correct for differences in survey efforts by collection site and collection equipment, the trap index (TI) was calculated. The TI was calculated by dividing the number of collected mosquito individuals by the total number of trap-nights actually operated at the corresponding site, where one trap-night was defined as operating one trap for one day.

### 2. Mosquito Species Identification and Pathogen Detection Testing

Collected mosquitoes were pretreated at −20℃ for 30 minutes or more, followed by classification and identification. The classified mosquitoes were pooled in groups of individuals according to species, collection period, and collection site. Mosquito tissues were uniformly homogenized using an automatic tissue homogenizer (homogenizer, FastPrep-24^TM^; MP Biomedicals), and RNA was extracted using a nucleic acid extraction kit (QIAamp Viral RNA Mini Kit; Qiagen). To confirm the carriage of flaviviruses in mosquitoes, the extracted RNA was subjected to a nucleic acid detection test using a multiplex real-time RT-PCR kit (Clear-MD^Ⓡ^ DENV/ZIKV/CHIKV/JEV/WNV/YFB Multiplex Real-Time RT-PCR Detection Kit; Invirustech). Amplification of flavivirus and CHIKV genes was confirmed through melting curves.

### 3. Utilization of Results

The surveillance results were uploaded to the integrated vector surveillance system (VectorNet) of the Infectious Disease Information System for each round, and the results were sent back to the quarantine stations.

## Results

### 1. Investigation of Mosquito Distribution by Quarantine Area in the Chungcheong Region in 2025

During the 9-month period from March to November 2025, a total of 8,393 mosquitoes across nine species were collected from nine sites within the quarantine areas of the Chungcheong region (Table 3). Regarding the distribution of potential vector species for major mosquito-borne infectious diseases, 5,647 individuals (67.3%) of *Culex pipiens* and 334 individuals (4.0%) of *Aedes vexans*, which are potential vectors of West Nile virus, were collected. In addition, 1,415 individuals (16.9%) of *Ae. albopictus*, a potential vector of dengue virus, Zika virus, and CHIKV, were collected. In particular, the collection numbers for *Cx. pipiens* and *Ae. albopictus* increased by approximately 2.5 times (152.7%) and 1.5 times (52.3%) from 2,235 and 929 individuals in 2024, respectively [8]. By quarantine area, the total number of collected individuals was the highest in Gunsan Port with 4,938 individuals (58.8%), followed by Daesan Port with 2,260 individuals (26.9%), Pyeongtaek-Dangjin Port with 1,150 individuals (13.7%), and Cheongju International Airport with 45 individuals (0.5%). Furthermore, consistent with the number of collected individuals, the number of collected species was the highest at Gunsan Port (9 species), followed by Daesan Port (8 species), Pyeongtaek-Dangjin Port (6 species), and Cheongju International Airport (6 species) (Table 3). Regarding the collected mosquito species by quarantine area, the proportion of *Cx. pipiens* was the highest in Gunsan Port and Pyeongtaek-Dangjin Port, whereas the proportion of *Ae. albopictus* was the highest in Daesan Port and Cheongju International Airport (Table 3). Next, the monthly mosquito occurrence by quarantine area according to the collection period was investigated (Table 4, Figure 1). In the Gunsan Port collection area, 1,675 individuals were collected in June, representing the highest monthly collection count, followed by 1,402 individuals in July. In the Daesan Port collection area, the number of collected individuals was high from May to September, with the highest monthly collection count in August at 665 individuals. At Pyeongtaek-Dangjin Port and Cheongju International Airport, the highest monthly collection counts were recorded in September (494 individuals) and August (19 individuals), respectively. Based on a total of 160 trap-nights, the species-specific TI was the highest for *Cx. pipiens* at 35.3 mosquitoes/trap-night, followed by *Ae. albopictus* at 8.8 mosquitoes/trap-night and *Ae. vexans* at 2.1 mosquitoes/trap-night (Table 5).

### 2. Investigation of Monthly Mosquito Occurrence by Quarantine Area in the Chungcheong Region in 2025

The occurrence status of mosquitoes by quarantine area according to the collection period was investigated. The monthly number of collected mosquitoes increased from April to July, reaching a peak of 2,098 individuals in July. Then, it decreased to 1,344 individuals in August, rose again to 1,783 individuals in September, and decreased in October and November. Regarding the monthly individual counts by collected species, *Cx. pipiens* was the most abundant in all months except May, when *Ae. vexans* was the most abundant with 309 individuals collected. The abundance of *Ae. albopictus* increased from June to September and showed a decreasing trend thereafter. *Ae. vexans* was not collected in March and April, but its count rapidly increased in May with 309 individuals collected, after which it decreased again, remaining at 10 or fewer individuals per month until October. For *Anopheles* spp., one individual was collected in June, followed by 223 individuals in July, showing the highest abundance, while *Ochlerotatus koreicus* was most collected in June with 146 individuals and decreased from July onwards.

### 3. Flavivirus Infection Investigation in Mosquitoes

We investigated the presence of infections with flaviviruses (dengue virus, yellow fever virus, West Nile virus, Zika virus, and Japanese encephalitis virus) and CHIKV in mosquitoes collected within the quarantine areas. Pooling up to 30 mosquitoes by species and region, a total of 8,393 mosquitoes in 500 pools were subjected to nucleic acid detection tests for the six viral species, and as a result, none of the target viruses were detected.

## Discussion

Recently, due to the increase in international exchange and overseas travel, as well as the impact of climate change, the risk of inflow of imported mosquito-borne infectious diseases, such as dengue fever, chikungunya fever, and Zika virus infection, into the ROK has been continuously increasing [7]. Accordingly, quarantine areas such as airports and ports are becoming increasingly important as crucial quarantine strongholds to detect the introduction of infectious disease vectors into the country at an early stage and block local community transmission [2].

This study aimed to analyze the results of mosquito surveillance conducted in quarantine areas of the Chungcheong region in 2025 to determine the species distribution, seasonal occurrence patterns of major vector mosquitoes, and their carrying of pathogens. As a result of the survey, a total of 8,393 mosquitoes across nine species were collected, and *Cx. pipiens* was identified as the dominant species, accounting for 67.3% (5,647 individuals) of the total collected individuals. *Cx. pipiens* is known as a major vector species of West Nile fever and is characterized by its ability to adapt well to urban sewers and artificial aquatic environments [9]. The predominant identification of *Cx. pipiens* in the quarantine areas of Gunsan Port and Pyeongtaek-Dangjin Port may be related to the urbanized environment around the ports. Conversely, *Ae. albopictus* was identified as the dominant species in the quarantine areas of Daesan Port and Cheongju International Airport, which appears to be because the green areas and tree distribution near the collection sites provided a suitable habitat for *Ae. albopictus* [9]. This result was consistent with previous studies showing that local environmental characteristics influence mosquito species composition [10]. In particular, *Ae. albopictus* accounted for 16.9% of the total collected individuals, emerging in June and showing a seasonal pattern of occurrence concentrated between July and September. Since *Ae. albopictus* is known as a key vector species capable of transmitting dengue fever, chikungunya fever, and Zika virus infection in the ROK, continuous surveillance within quarantine areas is necessary. The seasonal occurrence patterns observed in this study were similar to the results of previous studies conducted in domestic urban parks and residential areas, reconfirming the trend of population increase due to rising summer temperatures [11].

Meanwhile, in the Gunsan Port quarantine area, a large number of individuals were collected in June and July, followed by a decrease in August. The summer of 2025 was a period of repeated record-breaking heat waves and heavy rains, and these extreme weather events may have affected the mosquito population density by causing the loss or disturbance of larval habitats [12,13]. Therefore, when interpreting the mosquito occurrence patterns in quarantine areas in the future, it is necessary to consider not only temperature and precipitation but also various ecological factors, such as changes in the environment surrounding the collection sites.

As a result of performing nucleic acid detection tests for flaviviruses and CHIKV on the 8,393 collected mosquitoes, all were confirmed as negative. Considering the increase in patients with imported mosquito-borne infectious diseases and the expansion of international exchange, the potential risk of importation of mosquito-borne pathogens into the ROK in the future cannot be ruled out [14]. In particular, since *Ae. albopictus* and *Cx. pipiens* inhabiting the ROK are known to be capable of vectoring imported infectious diseases such as dengue fever and West Nile fever, continuous pathogen surveillance within quarantine areas is necessary. Because the present study analyzed vector surveillance results from quarantine areas in the Chungcheong region conducted over a single year in 2025, there are limitations in evaluating long-term occurrence trends. Another limitation was that quantitative analyses of environmental characteristics and meteorological factors for each collection site were not conducted. Nevertheless, this study is significant in that it systematically analyzed vector surveillance results from major quarantine areas in the Chungcheong region to investigate regional mosquito distribution characteristics and pathogen carrying status.

In conclusion, mosquito surveillance within quarantine areas in the Chungcheong region is considered an essential surveillance system for early detection and response to the risk of inflow of imported mosquito-borne infectious diseases into the ROK. The results of this study can be utilized as foundational data for establishing vector surveillance strategies and making effective quarantine policy decisions within quarantine areas, and are expected to contribute to strengthening response capabilities for imported infectious diseases through continuous surveillance and long-term data accumulation in the future.

## Figures and Tables

**Figure 1:**
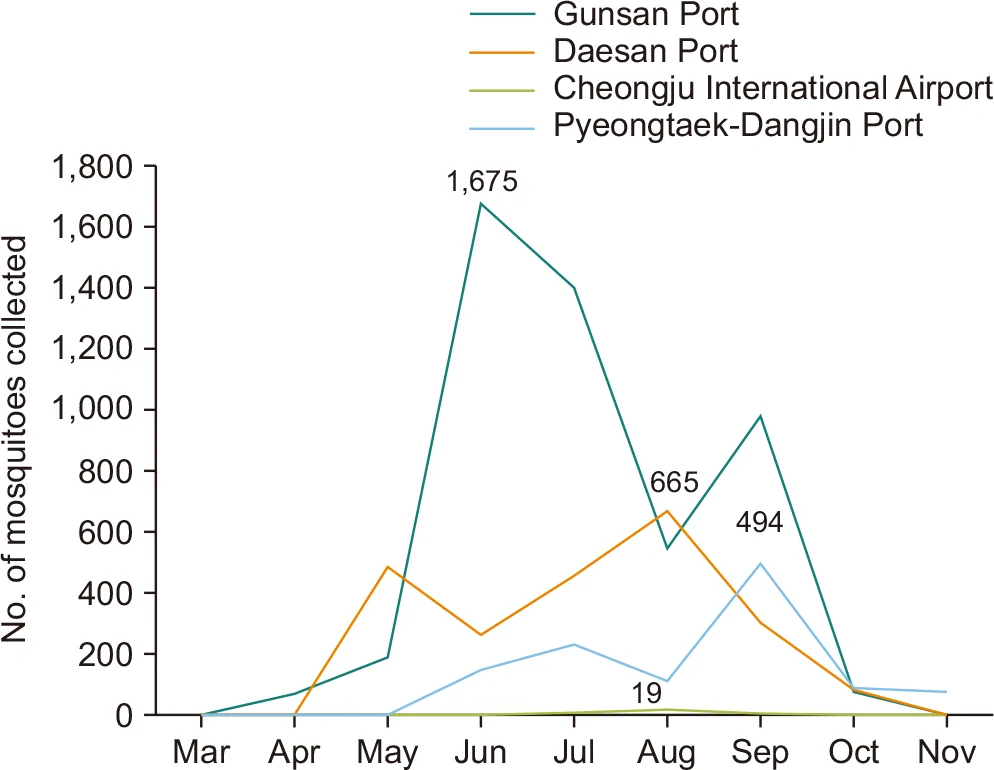
Number of mosquitoes collected by surveillance area per month, 2025

**Table 1:** Collection sites and equipment status by site

Quarantine zone	Collection site	Trap type
Gunsan Port	Gunsan Port Pier 1 (35.9736302, 126.6271004)	BG
	Gunsan Port Pier 2 (35.9704364, 126.6194242)	BG
	Janghang Port (36.0072673, 126.6958908)	BG
	Gunsan International Passenger Terminal (35.9779955, 126.6302741)	BG
Pyeongtaek-Dangjin Port	Pyeongtaek Port Marine Center (36.9577406, 126.8484458)	BG
	Pyeongtaek Coast Guard Station (36.9518404, 126.8468428)	BG
Daesan Port	Open space near the Daesan Branch Office (37.0126298, 126.4264755)	BG, gravid
	Daesan Port International Passenger Terminal (37.0134339, 126.4213430)	BG
Cheongju International Airport	Cheongju International Airport (36.7195899, 127.4932975)	BL, BG

BG=Biogents-sentinel trap; BL=black light trap.

**Table 2:** Monthly mosquito trap deployment schedule in quarantine zones in the Chungcheong region, 2025

Sampling round	Mar	Apr	May	Jun	Jul	Aug	Sep	Oct	Nov
Round 1	-	7	12	2	7	4	8	13	10
Round 2	17	21	26	16	21	18	22	27	-

Dates indicate trap deployment dates. Mosquitoes were collected 24 hours post-deployment. Sampling was conducted twice monthly, except in March and November, when it was conducted once. -=not performed.

**Table 3:** Species distribution of mosquitoes by quarantine area in the Chungcheong region, 2025

Species	No. of collected female mosquitoes									Total (%)
	Gunsan Port	Pyeongtaek- Dangjin Port	Daesan Port				Cheongju International Airport			
			Total	BG	Gravid trap		Total	BG	BL	
*Culex pipiens*	4,294	760	580	537	43		13	13	-	5,647 (67.28)
*Aedes albopictus*	206	350	835	796	39		24	24	-	1,415 (16.86)
*Aedes vexans*	7	5	320	315	5		2	2	-	334 (3.98)
*Anopheles* spp.	240	3	37	36	1		3	3	-	283 (3.37)
*Ochlerotatus koreicus*	93	21	116	116	-		1	1	-	231 (2.76)
*Ochlerotatus dorsalis*	10	-	189	173	16		-	-	-	199 (2.37)
*Armigeres subalbatus*	7	11	164	162	2		2	2	-	184 (2.19)
*Culex tritaeniorhynchus*	80	-	19	16	3		-	-	-	99 (1.18)
*Coquillettidia ochracea*	1	-	-	-	-		-	-	-	1 (0.01)
Total (%)	4,938 (58.8)	1,150 (13.7)	2,260 (26.9)	2,151 (25.6)	109 (1.3)		45 (0.5)	45 (0.5)	- (-)	8,393 (100)

BG=Biogents-sentinel trap; BL=black light trap.

**Table 4:** Monthly mosquito abundance by quarantine area in the Chungcheong region, 2025

Category		Mar	Apr	May	Jun	Jul	Aug	Sep	Oct	Nov	Total (%)
Gunsan Port	Total	-	68	189	1,675	1,402	548	977	79	-	4,938 (58.8)
	Pier 1	-	29	100	948	438	257	29	25	-	1,826 (21.8)
	Pier 2	-	21	10	238	84	171	908	15	-	1,447 (17.2)
	Janghang Port	-	9	45	6	672	85	1	33	-	851 (10.1)
	International Passenger Terminal	-	9	34	483	208	35	39	6	-	814 (9.7)
Pyeongtaek-Dangjin Port	Total	-	1	-	148	230	112	494	90	75	1,150 (13.7)
	Pyeongtaek Port Marine Center	-	-	-	72	86	87	240	31	7	523 (6.2)
	Pyeongtaek Coast Guard Station	-	1	-	76	144	25	254	59	68	627 (7.5)
Daesan Port	Total	-	3	485	263	455	665	305	83	1	2,260 (26.9)
	International Passenger Terminal	-	1	396	85	281	465	147	72	-	1,447 (17.2)
	Open space near the Daesan Branch Office (BG)	-	2	86	166	157	186	102	4	1	704 (8.4)
	Open space near the Daesan Branch Office (gravid)	-	-	3	12	17	14	56	7	-	109 (1.3)
Cheongju International Airport (1 site)		-	-	1	2	11	19	7	4	1	45 (0.5)
Total (%)		- (0)	72 (0.86)	675 (8.04)	2,088 (24.88)	2,098 (25.00)	1,344 (16.01)	1,783 (21.24)	256 (3.05)	77 (0.92)	8,393 (100)

Unit: number of collected female mosquitoes. BG=Biogents-sentinel trap; -=no mosquitoes collected (0 individuals).

**Table 5:** Number of mosquitoes collected per trap per night by species, 2025

Category	No. of collected female mosquitoes	Total trap-night	Trap indexa)
*Culex pipiens*	5,647	160	35.3
*Aedes albopictus*	1,415	160	8.8
*Aedes vexans*	334	160	2.1
*Anopheles spp.*	283	160	1.8
*Ochlerotatus koreicus*	231	160	1.4
*Ochlerotatus dorsalis*	199	160	1.2
*Armigeres subalbatus*	184	160	1.2
*Culex tritaeniorhynchus*	99	160	0.6
*Coquillettidia ochracea*	1	160	<0.1

a) Trap index=number of mosquitoes collected/total trap-nights; one trap-night was defined as one trap operated for one day.

## References

[1] World Health Organization (WHO). Global vector control response 2017–2030. WHO; 2017.

[2] World Health Organization (WHO). Vector surveillance and control at ports, airports, and ground crossings. WHO; 2016.

[3] Reinhold JM, Lazzari CR, Lahondère C. Effects of the environmental temperature on *Aedes aegypti* and *Aedes albopictus* mosquitoes: a review. Insects 2018;9:158. 10.3390/insects9040158 30404142 c222239174e549c9bf65fc64475e6928 PMC6316560

[4] Mordecai EA, Caldwell JM, Grossman MK, et al. Thermal biology of mosquito-borne disease. Ecol Lett 2019;22:1690-708. 10.1111/ele.13335 31286630 PMC6744319

[5] Korea Disease Control and Prevention Agency (KDCA). Infectious disease portal: dengue fever statistics [Internet]. KDCA [cited 2026 Jun 16]. Available from: https://dportal.kdca.go.kr/pot/index.do

[6] Korea Disease Control and Prevention Agency (KDCA). Global infectious disease outbreak update, No. 30 [Internet]. KDCA; 2025 [cited 2026 Jun 16]. Available from: https://dportal.kdca.go.kr/pot/bbs/BD_selectBbs.do?q_bbsSn=1009&q_bbsDocNo=20250808145847032&q_clsfNo=-1

[7] Park H, Kwon PS. Monitoring and distribution of mosquitoes in the downtown areas and migratory bird habitats in the Jeonbuk region, 2021. Korean J Clin Lab Sci 2022;54:142-8. 10.15324/kjcls.2022.54.2.142 356e8d3157494a02afe792e796ea8520

[8] Korea Disease Control and Prevention Agency Press Release (December 10 2025). Mosquito and Vibrio surveillance to prevent the introduction of imported infectious diseases. [cited 2026 Jul 13]. Available from: https://www.kdca.go.kr/kdca/2847/subview.do?enc=Zm5jdDF8QEB8JTJGYmJzJTJGa2RjYSUyRjQxJTJGMjE1NTIzJTJGYXJ0Y2xWaWV3LmRvJTNGcGFzc3dvcmQlM0QlMjZyZ3NCZ25kZVN0ciUzRCUyNmZpbmRPcG53cmQlM0QlMjZmaW5kV29yZCUzRCVFQiVBQSVBOCVFQSVCOCVCMCUyNnJnc0VuZGRlU3RyJTNEJTI2ZmluZFR5cGUlM0RzaiUyNmZpbmRDbFNlcSUzRCUyNnBhZ2UlM0QxJTI2

[9] Lee DK. Ecological characteristics and current status of infectious disease vectors in South Korea. J Korean Med Assoc 2017;60:458-67. 10.5124/jkma.2017.60.6.458

[10] Ferraguti M, Martínez-de la Puente J, Roiz D, Ruiz S, Soriguer R, Figuerola J. Effects of landscape anthropization on mosquito community composition and abundance. Sci Rep 2016;6:29002. 10.1038/srep29002 27373794 PMC4931447

[11] Hwang MJ, Kim JH, Kim HC, et al. Temporal trend of *Aedes albopictus* in local urban parks of the Republic of Korea. J Med Entomol 2020;57:1082-9. 10.1093/jme/tjaa039 32185384

[12] Korea Meteorological Administration Press Release (September 4 2025). Climate characteristics of summer 2025. [cited 2026 Jun 16]. Available from: https://www.kma.go.kr/kma/news/press_01.jsp?bid=press&mode=view&num=1194521&page=1&&from=2025-09-01&to=2025-09-15

[13] Oh HS, Seo J, Ju JW, Lee HI. Surveillance results of Japanese encephalitis vector mosquitoes in the Republic of Korea in 2025. Public Health Wkly Rep 2026;19:839-58. 10.56786/PHWR.2026.19.20.1 PMC13228900 42238327

[14] Oh HS. Travel medicine clinic network for the prevention and early detection of imported infectious diseases. Public Health Wkly Rep 2025;18:1411-30. 10.56786/PHWR.2025.18.38.1 PMC12479904 41334093

