# Supplementary material for: Mosquito Surveillance in Quarantine Areas of Ports and Airports in the Chungcheong Region, Republic of Korea, 2025
Source: Public Health Wkly Rep. 2026 Nov 5;19(42):1770–86. doi: 10.56786/PHWR.2026.19.42.3 (PMC13740382; doi:10.56786/PHWR.2026.19.42.3)
Supplement: Translation

# Translation: 2025년 충청권 공ㆍ항만 검역구역 내 감염병 매개 모기 분포 조사

**Authors:** 별희 임, 영실 윤, 준형 장, 소연 김, 보람 김, 준호 전

**Affiliations:** 질병관리청 충청권질병대응센터 진단분석과

**Keywords:** 모기매개감염병, 매개체감시, 플라비바이러스, 검역

## Abstract

**목적:**

질병관리청 충청권질병대응센터에서는 해외 감염병 매개 모기의 국내유입 및 지역사회 확산 방지를 위해 충청권 공ㆍ항만 검역구역 내 감염병 매개 모기의 분포와 모기 내 병원체 보유 여부를 조사하였다.

**방법:**

충청권 공ㆍ항만 검역구역(군산항, 평택-당진항, 대산항, 청주국제공항) 내 9개 지점에서 2025년 3월부터 11월까지 9개월간 매월 2회(3월과 11월은 월 1회) 유문등, Biogents-sentinel 트랩, 그리고 gravid 트랩을 이용하여 모기를 채집하였다. 채집된 모기는 분류동정 후 플라비바이러스 및 치쿤구니야바이러스 감염 여부를 유전자 검출검사를 이용하여 조사하였다.

**결과:**

총 9종, 8,393마리의 모기가 충청권 검역구역에서 채집되었다. 모기종별로는 빨간집모기가 전체의 67.3%인 5,647마리가 채집되어 가장 높은 비율을 차지하였으며 흰줄숲모기(1,415마리, 16.9%), 금빛숲모기 (334마리, 4.0%), 얼룩날개모기류 (283마리, 3.4%), 한국숲모기 (231마리, 2.8%) 순으로 채집되었다. 월별로는 7월에 가장 많은 개체수가 채집되었고 검역구역별로는 군산항 채집지점에서 전체 채집모기의 58.8% (4,938마리)가 채집되어 가장 많은 개체수가 채집되었다. 채집모기를 대상으로 실시한 플라비바이러스 및 치쿤구니야바이러스 유전자 검출검사 결과는 모두 음성이었다.

**결론:**

충청권 검역구역 내 감염병 매개 모기 감시 결과 이집트숲모기와 같은 해외 모기종과 모기 내 병원체 감염은 확인되지 않았지만 웨스트나일바이러스의 잠재적인 매개종인 빨간집모기와 뎅기바이러스, 지카바이러스, 치쿤구니야바이러스 등의 잠재적인 매개종인 흰줄숲모기가 전년(빨간집모기 2,235마리, 흰줄숲모기 929마리) 대비 약 2.5배(152.7%)와 약 1.5배(52.3%) 증가하였다. 따라서 검역구역 내 지속적인 매개모기 감시 결과는 해외 감염병 국내유입 위험의 조기 탐지와 효과적인 검역 및 방역체계 구축을 위한 기초자료로 활용될 수 있을 것으로 기대된다.

**핵심요약**

**① 이전에 알려진 내용은?**

모기는 다양한 감염병을 전파하는 대표적인 매개체로 알려져 있다. 특히 뎅기열, 치쿤구니야열, 지카바이러스감염증 및 웨스트나일열과 같은 해외유입 모기매개감염병은 국제교류 증가와 기후변화로 발생 위험이 증가하고 있으며, 국내에 서식하는 흰줄숲모기와 빨간집모기 등도 뎅기열, 웨스트나일열 등의 해외유입 모기매개감염병의 매개가 가능한 것으로 알려져 있다.

**② 새로이 알게 된 내용은?**

2025년 충청권 검역구역 내 매개체 감시사업 결과 총 9종 8,393마리의 모기가 채집되었으며 빨간집모기가 우점종으로 확인되었다. 채집된 모기를 대상으로 실시한 플라비바이러스 및 치쿤구니야바이러스 유전자 검출검사 결과는 모두 음성이었다.

**③ 시사점은?**

최근 중국 등 인접국가에서 치쿤구니야열 발생이 보고되는 등 해외유입 모기매개감염병의 위험이 지속되고 있다. 검역구역 내 감염병 매개모기 감시는 해외유입 감염병의 국내유입 위험을 조기에 파악하고 대응하기 위한 기초자료를 제공함으로써 효과적인 검역 및 방역체계구축에 기여할 수 있다.

## 서 론

최근 국제교류와 해외여행 증가에 따라 해외유입 감염병의 국내유입 위험이 지속적으로 증가하고 있다. 특히 뎅기열, 치쿤구니야열, 지카바이러스감염증 및 웨스트나일열과 같은 모기매개감염병은 감염자와 매개체의 이동을 통해 국가 간 전파가 가능하여 국제 공중보건의 주요 위협요인으로 인식되고 있다[1,2].

기후변화는 모기매개감염병의 발생 및 확산에 영향을 미치는 주요 요인으로 알려져 있다. 기온 상승과 강수 양상의 변화는 모기의 생존, 번식 및 계절적 발생 양상에 영향을 미치며 일부 모기종의 서식 범위 확대와 발생 시기 변화를 초래할 수 있다[3,4]. 이에 따라 세계보건기구(World Health Organization, WHO)는 모기매개감염병 예방을 위한 감시체계 강화와 지속적인 매개체 관리의 중요성을 강조하고 있다[2].

국내에서는 해외유입 뎅기열 환자가 지속적으로 보고되고 있으며[5] 2025년 중국 광둥성에서 치쿤구니야열 유행이 보고되는 등 해외유입 모기매개감염병의 국내유입 위험이 지속되고 있다[6]. 치쿤구니야열과 뎅기열은 국내에 서식하는 흰줄숲모기에 의해 전파가 가능한 것으로 알려져 있어 해외 감염환자와 해외유입감염 매개체를 통한 국내유입 가능성에 대한 지속적인 평가와 검역구역에서의 해외유입 매개체 감시의 중요성이 더욱 커지고 있다[7].

WHO는 공항과 항만 등 국제교류가 이루어지는 지역에서의 매개체 감시 역량 강화를 권고하고 있다[2]. 국내에서도 「검역법」 및 검역업무지침에 따라 검역구역을 대상으로 감염병 매개체 감시사업을 수행하고 있다. 해외유입 감염병의 국내유입을 조기에 탐지하고 확산을 예방하기 위한 노력을 지속하고 있다.

충청권은 청주국제공항과 군산항, 평택-당진항 및 대산항 등 주요 국제교류 거점이 위치한 지역으로 모기매개 해외감염병 및 감염병 매개체의 국내유입 가능성이 높은 지역이다. 따라서 본 연구에서는 2025년 충청권 검역구역에서 채집된 모기의 종 분포와 계절적 발생 양상을 분석하고 모기 내 플라비바이러스 및 치쿤구니야바이러스 보유 여부를 조사함으로써 검역구역 내 해외유입 감염병 매개체의 유입 위험을 평가하고 향후 검역구역 내 매개체 감시 전략 수립을 위한 과학적 근거를 마련하고자 하였다.

## 방 법

### 1. 모기 채집

해외유입 감염병 매개모기와 모기 내 병원체 감시를 위해 충청권 검역구역 내 9개 지점(군산항 4개 지점, 평택-당진항 2개 지점, 대산항 2개 지점, 청주국제공항 1개 지점)에서 3월부터 11월까지 월 2회(3월과 11월은 월 1회) 모기 채집을 실시하였다(표 1, 2). 채집지점은 검역구역 내 화물 선적장 또는 여행객의 이동경로와 같은 사람의 왕래가 많고, 하수구 또는 물고임이 있으며 나무와 수풀이 있는 환경으로 선택하였다. 모기 채집을 위한 장비는 유문등(전기포충기; 바이오트랩)과 Biogents (BG)-sentinel 트랩(BG-sentinel trap; Biogents), gravid 트랩(CDC gravid trap; John W.Hock Company)을 사용하였다(표 1). 대산지소 청사 근처 공터에는 BG-sentinel 트랩과 gravid 트랩이 서로 영향을 미치지 않도록 10 m 이상의 거리를 두고 gravid 트랩을 설치하였다. 청주국제공항에서는 4월까지 유문등을 사용하고 5월부터 BG-sentinel 트랩을 사용하였다. 모기유인제로는 드라이아이스와 루어를 사용하였다. 채집지점 및 채집장비별 조사 노력의 차이를 보정하기 위하여 트랩지수(trap index, TI)를 산출하였다. TI는 채집된 모기 개체수를 해당 지점에서 실제 운영된 총 trap-night로 나누어 계산하였으며, 1 trap-night는 1개의 트랩을 1일간 운영한 것으로 정의하였다.

### 2. 모기종 동정 및 병원체 검출검사

채집된 모기는 –20℃에서 30분 이상 전처리 후 분류동정하였다. 분류된 모기는 종별, 채집시기, 채집장소에 따라 최대 30마리까지 취합하여 자동조직균질기(homogenizer, FastPrep-24^TM^; MP Biomedicals)를 이용하여 모기조직을 균일하게 파쇄한 후 핵산추출키트(QIAamp Viral RNA Mini Kit; Qiagen)를 이용하여 RNA를 추출하였다. 추출한 RNA는 모기 내 플라비바이러스 보유 여부를 확인하기 위해 다중 실시간 역전사중합효소연쇄반응용 키트(Clear-MD^Ⓡ^ DENV/ZIKV/CHIKV/JEV/WNV/YFB Multiplex Real-Time RT-PCR Detection Kit; Invirustech)를 이용하여 유전자 검출검사를 수행하였다. 플라비바이러스와 치쿤구니야바이러스 유전자 증폭 여부는 융해곡선(melting curve)을 통해 확인하였다.

### 3. 결과활용

감시 결과는 회차별로 방역통합정보시스템의 매개체 통합감시시스템(VectorNet)에 업로드하고 검역소에 결과를 회신하였다.

## 결 과

### 1. 2025년 충청권 검역구역별 모기 분포 조사

2025년 3월부터 11월까지 9개월간 충청권 검역구역 내 9개 지점에서 채집된 모기는 9종 8,393마리였다(표 3). 주요 모기매개감염병별 잠재적 매개종의 분포를 살펴보면 웨스트나일바이러스의 잠재적 매개종인 빨간집모기와 금빛숲모기는 각각 5,647마리(67.3%)와 334마리(4.0%)가 채집되었다. 또한 뎅기바이러스, 지카바이러스 및 치쿤구니야바이러스 등의 잠재적 매개종인 흰줄숲모기는 1,415마리(16.9%)가 채집되었다. 특히 빨간집모기와 흰줄숲모기의 채집량은 2024년 각각 2,235마리와 929마리에서 약 2.5배(152.7%)와 약 1.5배(52.3%) 증가하였다[8]. 검역구역별 총 채집 개체수는 군산항에서 4,938마리(58.8%)로 가장 많았으며, 대산항 2,260마리(26.9%), 평택-당진항 1,150마리(13.7%), 청주국제공항 45마리(0.5%) 순이었다. 또한 채집 종의 수도 채집 개체수와 같이 군산항(9종)이 가장 많았으며 대산항(8종), 평택-당진항(6종)과 청주국제공항(6종) 순으로 확인되었다(표 3). 검역구역별 모기 채집종은 군산항과 평택-당진항에서는 빨간집모기의 비율이 가장 높았으며 대산항과 청주국제공항에서는 흰줄숲모기의 비율이 가장 높았다(표 3). 그 다음으로는 채집시기에 따른 검역구역별 월별 모기 발생현황을 조사하였다(표 4, 그림 1). 군산항 채집지역에서는 6월에 1,675마리가 채집되어 월별 채집 개체수가 가장 많았으며 다음으로 7월에 1,402마리가 채집되었다. 대산항 채집지역에서는 5월부터 9월까지 채집 개체수가 많았으며 그중 8월에 665마리가 채집되어 월별 채집 개체수가 가장 많았다. 평택-당진항과 청주국제공항에서는 각각 9월에 494마리, 8월에 19마리가 채집되어 각 지역의 월별 채집 개체수가 가장 많았다. 총 160 trap-nights를 기준으로 산출한 종별 TI는 빨간집모기가 35.3마리/trap-night로 가장 높았으며, 흰줄숲모기 8.8마리/trap-night, 금빛숲모기 2.1마리/trap-night 순이었다(표 5).

### 2. 2025년 충청권 검역구역별 월별 모기 발생 조사

채집시기에 따른 검역구역별 모기 발생 현황을 조사하였다. 채집 모기의 월별 개체수는 4월부터 7월까지 증가하였으며, 7월에 2,098마리로 가장 많았다. 이후 8월에 1,344마리로 감소하였다가 9월에 1,783마리로 다시 증가한 후 10월과 11월에는 감소하였다. 채집종별 월별 개체수는 5월을 제외하고는 빨간집모기가 가장 많았으며 5월에는 금빛숲모기가 309마리 채집되어 가장 많은 개체수가 채집되었다. 흰줄숲모기는 6월부터 9월까지 개체수가 증가하였으며, 그 이후 감소하는 경향을 보였다. 금빛숲모기는 3월과 4월에는 채집되지 않다가 5월에 개체수가 급격히 증가하여 309마리가 채집된 후 다시 감소하여 10월까지 10마리 이내의 채집 개체수를 보였다. 얼룩날개모기류는 6월에 1마리가 채집된 후 7월에 223마리가 채집되어 가장 많은 개체수를 보였으며 한국숲모기는 6월에 146마리로 가장 많이 채집되었으며 7월부터 감소하였다.

### 3. 모기 내 플라비바이러스 감염조사

검역구역 내 채집 모기에서의 플라비바이러스(뎅기바이러스, 황열바이러스, 웨스트나일바이러스, 지카바이러스, 일본뇌염바이러스) 및 치쿤구니야바이러스 감염 여부를 조사하였다. 모기종별, 지역별 최대 30마리씩 취합하여 총 8,393마리 500개의 실험군에서 6종 바이러스에 대한 유전자 검출검사를 수행한 결과 해당 바이러스는 모두 검출되지 않았다.

## 논 의

최근 국제교류와 해외여행 증가, 기후변화의 영향으로 뎅기열, 치쿤구니야열, 지카바이러스감염증 등 해외유입 모기매개감염병의 국내유입 위험이 지속적으로 증가하고 있다[7]. 이에 따라 공항과 항만 등 검역구역은 감염병 매개체의 국내유입을 조기에 탐지하고 지역사회 확산을 차단하기 위한 중요한 방역 거점으로서 그 역할이 더욱 중요해지고 있다[2].

본 연구는 2025년 충청권 검역구역에서 수행된 모기 감시 결과를 분석하여 주요 매개모기의 종 분포와 계절적 발생 양상, 그리고 병원체 보유 여부를 확인하고자 하였다. 조사 결과 총 9종 8,393마리의 모기가 채집되었으며 빨간집모기가 전체 채집 개체의 67.3% (5,647마리)를 차지하여 우점종으로 확인되었다. 빨간집모기는 웨스트나일열의 주요 매개종으로 알려져 있으며, 도시지역의 하수구나 인공 수계 환경에 잘 적응하는 특성을 가진다[9]. 군산항과 평택-당진항 검역구역에서 빨간집모기가 우세하게 확인된 것은 항만 주변의 도시화된 환경과 관련이 있을 가능성이 있다. 반면 대산항과 청주국제공항 검역구역에서는 흰줄숲모기가 우점종으로 확인되었는데 이는 채집지 인근의 녹지환경과 수목 분포가 흰줄숲모기의 서식에 적합한 환경을 제공한 결과로 보인다[9]. 이러한 결과는 주변 환경 특성이 모기 종 구성에 영향을 미친다는 기존 연구 결과와도 일치한다[10]. 특히 흰줄숲모기는 전체 채집 개체의 16.9%를 차지하였으며 6월부터 출현하여 7–9월에 집중적으로 발생하는 계절적 양상을 나타냈다. 흰줄숲모기는 국내에서 뎅기열, 치쿤구니야열 및 지카바이러스감염증 전파 가능성이 있는 주요 매개종으로 알려져 있어 검역구역 내 지속적인 감시가 필요하다. 본 연구에서 확인된 계절적 발생 양상은 국내 도시공원과 주거지역을 대상으로 수행된 선행연구 결과와 유사하였으며, 여름철 기온 상승에 따른 개체수 증가 경향을 재확인하였다[11].

한편 군산항 검역구역에서는 6–7월에 많은 개체수가 채집되다가 8월에 감소하는 양상이 관찰되었다. 2025년 여름철은 기록적인 폭염과 집중호우가 반복된 시기로 이러한 극한 기상현상은 유충 서식처의 유실이나 교란을 유발하여 모기 개체군 밀도에 영향을 미쳤을 가능성이 있다[12,13]. 따라서 향후 검역구역 내 모기 발생 양상을 해석할 때에는 기온과 강수량뿐 아니라 채집지 주변 환경 변화 등 다양한 생태학적 요인을 함께 고려할 필요가 있다.

채집된 모기 8,393마리를 대상으로 플라비바이러스와 치쿤구니야바이러스 유전자 검출검사를 수행한 결과 모두 음성으로 확인되었다. 해외유입 모기매개감염병 환자의 증가와 국제교류 확대를 고려할 때 향후 모기매개감염병 병원체의 국내유입 가능성을 배제할 수는 없다[14]. 특히 국내에 서식하는 흰줄숲모기와 빨간집모기는 뎅기열, 웨스트나일열 등의 해외유입 감염병을 매개할 수 있는 것으로 알려져 있어 검역구역 내 지속적인 감시가 필요하다. 본 연구는 2025년 한 해 동안 수행한 충청권 검역구역에서의 매개체 감시 결과를 분석하였기 때문에 장기적인 발생 경향을 평가하는 데에는 한계가 있다. 또한 채집지점별 환경 특성 및 기상요인에 대한 정량적 분석이 수행되지 못한 제한점이 있다. 그럼에도 불구하고 본 연구는 충청권 주요 검역구역에서 수행된 매개체 감시 결과를 체계적으로 분석하여 지역별 모기 분포 특성과 병원체 보유 현황을 조사했다는 점에서 의의가 있다.

결론적으로 충청권 검역구역 내 모기 감시는 해외유입 모기매개감염병의 국내유입 위험을 조기에 탐지하고 대응하기 위한 필수적인 감시체계로 판단된다. 본 연구 결과는 검역구역 내 매개체 감시 전략 수립과 효과적인 방역정책 결정의 기초자료로 활용될 수 있으며, 향후 지속적인 감시와 장기적인 자료 축적을 통해 해외유입 감염병 대응 역량 강화에 기여할 것으로 기대된다.

## Figures and Tables

**그림 1:** 2025년 채집지역별 월별 모기 채집 개체수

**표 1:** 채집지점 및 지점별 장비 현황

검역구역	채집지점	채집장비
군산항	군산항 1부두(35.9736302, 126.6271004)	BG
	군산항 2부두(35.9704364, 126.6194242)	BG
	장항항(36.0072673, 126.6958908)	BG
	군산항 국제여객터미널(35.9779955, 126.6302741)	BG
평택-당진항	평택항 마린센터(36.9577406, 126.8484458)	BG
	평택해양경찰서 청사(36.9518404, 126.8468428)	BG
대산항	대산지소 청사 근처 공터(37.0126298, 126.4264755)	BG, gravid
	대산항 국제여객터미널(37.0134339, 126.4213430)	BG
청주국제공항	청주국제공항(36.7195899, 127.4932975)	유문등, BG

BG=Biogents-sentinel trap.

**표 2:** 2025년 충청권 검역구역에서의 월별 모기 채집 트랩 설치 일정

회차	3월	4월	5월	6월	7월	8월	9월	10월	11월
1회차	-	7	12	2	7	4	8	13	10
2회차	17	21	26	16	21	18	22	27	-

표에 제시된 날짜는 트랩 설치일이며, 설치 24시간 후 채집된 모기를 수거함. 채집은 월 2회, 3월과 11월에는 월 1회 실시. -=미실시.

**표 3:** 2025년 충청권 검역구역별 모기종 분포

종 류	채집 개체수(마리)									합계(%)
	군산항	평택-당진항	대산항				청주국제공항			
			합계	BG	Gravid 트랩		합계	BG	유문등	
빨간집모기	4,294	760	580	537	43		13	13	-	5,647 (67.28)
흰줄숲모기	206	350	835	796	39		24	24	-	1,415 (16.86)
금빛숲모기	7	5	320	315	5		2	2	-	334 (3.98)
얼룩날개모기류	240	3	37	36	1		3	3	-	283 (3.37)
한국숲모기	93	21	116	116	-		1	1	-	231 (2.76)
등줄숲모기	10	-	189	173	16		-	-	-	199 (2.37)
큰검정들모기	7	11	164	162	2		2	2	-	184 (2.19)
작은빨간집모기	80	-	19	16	3		-	-	-	99 (1.18)
노랑늪모기	1	-	-	-	-		-	-	-	1 (0.01)
합계(%)	4,938 (58.8)	1,150 (13.7)	2,260 (26.9)	2,151 (25.6)	109 (1.3)		45 (0.5)	45 (0.5)	- (-)	8,393 (100)

BG=Biogents-sentinel trap.

**표 4:** 2025년 충청권 검역구역별 월별 모기 발생 현황

구 분		3월	4월	5월	6월	7월	8월	9월	10월	11월	합계(%)
군산항	합계	-	68	189	1,675	1,402	548	977	79	-	4,938 (58.8)
	1부두	-	29	100	948	438	257	29	25	-	1,826 (21.8)
	2부두	-	21	10	238	84	171	908	15	-	1,447 (17.2)
	장항항	-	9	45	6	672	85	1	33	-	851 (10.1)
	국제여객터미널	-	9	34	483	208	35	39	6	-	814 (9.7)
평택-당진항	합계	-	1	-	148	230	112	494	90	75	1,150 (13.7)
	평택항 마린센터	-	-	-	72	86	87	240	31	7	523 (6.2)
	평택해양경찰서 청사	-	1	-	76	144	25	254	59	68	627 (7.5)
대산항	합계	-	3	485	263	455	665	305	83	1	2,260 (26.9)
	국제여객터미널	-	1	396	85	281	465	147	72	-	1,447 (17.2)
	대산지소청사 근처 공터(BG)	-	2	86	166	157	186	102	4	1	704 (8.4)
	대산지소청사 근처 공터(gravid)	-	-	3	12	17	14	56	7	-	109 (1.3)
청주국제공항(1지점)		-	-	1	2	11	19	7	4	1	45 (0.5)
합계(%)		- (0)	72 (0.86)	675 (8.04)	2,088 (24.88)	2,098 (25.00)	1,344 (16.01)	1,783 (21.24)	256 (3.05)	77 (0.92)	8,393 (100)

단위: 채집 개체수(마리). BG=Biogents-sentinel trap; -=모기가 채집되지 않음(0마리).

**표 5:** 2025년 모기 종별 트랩당 1일 채집 개체수

구 분	채집 개체수(마리)	총 trap-nights	트랩지수(TI)a)
빨간집모기	5,647	160	35.3
흰줄숲모기	1,415	160	8.8
금빛숲모기	334	160	2.1
얼룩날개모기류	283	160	1.8
한국숲모기	231	160	1.4
등줄숲모기	199	160	1.2
큰검정들모기	184	160	1.2
작은빨간집모기	99	160	0.6
노랑늪모기	1	160	<0.1

^a)^트랩지수(trap index, TI)=채집 개체수/총 trap-nights; 1 trap-night는 1개의 트랩을 1일간 운영한 것으로 정의함.
